# Transjugular intravascular ultrasound for the evaluation of hepatic vasculature and parenchyma in patients with chronic liver disease

**DOI:** 10.1186/1756-0500-5-77

**Published:** 2012-01-27

**Authors:** Andrea De Gottardi, Pierre-Frédéric Keller, Antoine Hadengue, Emile Giostra, Laurent Spahr

**Affiliations:** 1 Division of Gastroenterology and Hepatology, University Hospitals and Faculty of Medicine, Geneva, Switzerland; 2 Interventional Cardiology, University Hospitals and Faculty of Medicine, Geneva, Switzerland; 3 Hepatology Research Group, Department of Clinical Research, University of Berne, Murtenstrasse 35, 3010 Berne, Switzerland; 4 University Clinic of Visceral Surgery and Medicine, Inselspital, 3010 Berne, Switzerland

**Keywords:** Imaging, Intracardiac echography, Liver, Cirrhosis, Portal hypertension, Hepatocellular carcinoma

## Abstract

**Background:**

The evaluation of the hepatic parenchyma in patients with chronic liver disease is important to assess the extension, localization and relationship with adjacent anatomical structures of possible lesions. This is usually performed with conventional abdominal ultrasound, CT-scan or magnetic resonance imaging. In this context, the feasibility and the safety of intravascular ultrasound in the liver have not been assessed yet.

**Methods:**

We tested the safety and performance of an intracardiac echography (ICE) catheter applied by a transjugular approach into the hepatic veins in patients with chronic liver disease undergoing hepatic hemodynamic measurements.

**Results:**

Five patients were enrolled in this pilot study. The insertion of the ICE catheter was possible into the right and middle, but not into the left hepatic vein. The position of the ICE was followed using fluoroscopy and external conventional ultrasound. Accurate imaging of focal hepatic parenchymal lesions, Doppler ultrasound of surrounding blood vessels and assessment of liver surface and ascites were achieved without complications.

**Conclusions:**

This study demonstrated that a diagnostic approach using an ICE device inserted in the hepatic veins is feasible, safe and well tolerated. However, it remains for the moment only an experimental investigative tool. Whether ICE adds further information regarding parenchymal lesions and associated vascular alterations as compared to other techniques, needs additional investigation.

## Background

Assessment of disease extent is an important process in the management of patients with chronic liver disease and suspected parenchymal or vascular lesions [1-3]. The spatial relationship between lesions and their surrounding anatomical structures - portal vein branches, hepatic veins, bile ducts and liver capsule - is of particular importance for prognosis and choice of treatment.

For instance in hepatocellular carcinoma (HCC), the size and the number of lesions represent the most critical information, impacting therapeutic options, that include surgical or radiofrequency ablation, transarterial chemoembolization, or liver transplantation. Patients with a single HCC nodule can be treated by surgical resection provided that this procedure is technically feasible, that the Child-Pugh stage is A, that the serum total bilirubin level is normal, that there is no vascular invasion and that the hepatic venous pressure gradient (HVPG) is 10 mmHg or less [4]. For the evaluation of these preoperative conditions, precise imaging techniques like abdominal ultrasonography, spiral computer tomography and magnetic resonance imaging are currently widely used and provide excellent results [5]. The measurement of the free and wedged hepatic pressure through the transjugular route represents the gold standard method [6] to assess portal pressure and to decide whether a liver resection is possible [4].

Although surgical resection is the mainstay of curative treatment for HCC, long-term survival remains unsatisfactory, because tumor recurrence is the main cause of long-term death [7]. Clinical predictors of outcome include the presence of satellite nodules and vascular invasion [8]. The currently available imaging techniques (CT-scan, magnetic resonance imaging and external ultrasound) can hardly diagnose small HCC and reach a sensitivity not better than 64% for lesions of less than 10 mm in diameter [9]. The availability of imaging techniques that may identify minor satellite lesions and invasion of small diameter vessels would improve the preoperative assessment of early HCC.

For these reasons, percutaneous transvenous ultrasound may represent a complementary novel technique for the assessment of HCC. So far this technique has been used only for intracardiac echography (ICE), useful during electrophysiological mapping and ablation procedures for guiding interatrial septal puncture, and for the positioning of closure devices in patients with atrial septal defects [10,11]. The use of ICE through the hepatic veins allows to assess the anatomy of the liver parenchyma and the vessels and to evaluate the vascular flow by using the Doppler technique. ICE has never been reported for the imaging of the cirrhotic liver and HCC.

Consequently, the aim of this pilot study was to investigate the feasibility and safety of this procedure and to evaluate the quality of images that can be obtained with this technique.

## Methods

Cirrhotic patients addressed to the gastroenterology and hepatology division for the diagnostic work-up of suspected or proven HCC on other imaging techniques were eligible for this study. Transjugular liver biopsy and hepatic hemodynamic measurements were part of this work-up. Participants received a light sedation with 25 mg of meperidine intravenously.

**Figure 1:**
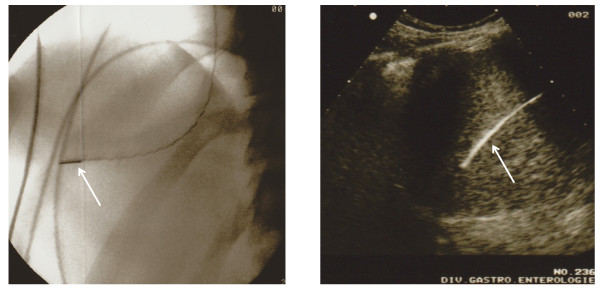
**Imaging of the liver by intrahepatic use of an intracardiac echography catheter (ICE)**. The position of the ICE catheter, here in the right hepatic vein, was assessed by conventional X-ray (left panel, white arrow) and by conventional abdominal ultrasound (right panel, white arrow).

Under sterile conditions, the right internal jugular vein was catheterized under local anesthesia (1% lidocaine) using the Seldinger technique and a 9 French introducer (Maxxim Medical, Europe) was inserted. All procedures were then performed under radiologic guidance using a C-arm X-ray amplifier and controlled by conventional abdominal ultrasound (Figure 1). Hemodynamic measurements in the right or middle hepatic veins were performed by using a 8 French curved catheter (Cordis, Europe) in free and wedged position connected to a pressure transducer. Thereafter, liver biopsy was performed according to a standardized protocol. Clinical vital data including systemic arterial pressure, pulse rate, and oxygen saturation were monitored throughout the procedure.

Next, the single-use intracardiac echography catheter (AcuNav 8 F, 5.5-10 MHz, Acuson) was introduced in sterile conditions in the hepatic veins under fluoroscopic control. The AcuNav catheter tip was steered by the operator into the chosen direction by external control knobs. The middle and right hepatic veins were catheterized in each case and the position of the device was also verified by transabdominal external ultrasound.

The intracardiac echography catheter was then rotated clock- and anti-clockwise to obtain a 360-degree view around. The ultrasound frequency of the catheter was 7.5 MHz for all images and the depth of liver parenchyma explored was adjusted for maximal resolution. The signals from the longitudinal side-fire imaging plane were visualized and recorded using an Acuson Sequoia C512 echocardiography platform. Vessels, parenchyma and possible lesions were also studied using color Doppler mode signals.

At the end of the procedure the catheter and the introducer were withdrawn and the puncture site was briefly compressed for hemostasis. Patients were then asked about pain, discomfort or other subjective problems that occurred during this procedure.

Because this was a pilot study, at the time of ICE the investigators were not blinded to the presence and localization of hepatic focal lesions identified by 3-phase CT-scan or magnetic resonance imaging.

The study protocol was approved by the committee of the University Hospitals of Geneva (protocol approval number 07-063) and patients gave their written consent for the participation in this study.

## Results

We report here the data from intravascular ultrasound examinations in 5 patients with hepatocellular carcinoma using the intracardiac echographic technique. The main clinical characteristics of these patients are presented in Table 1.

**Table 1:** Patients' characteristics

Patient	Age (Y)	Gender	Etiology of chronic liver disease	Child-Pugh score (points)	Fibrosis score (METAVIR)	MELD score	Clinical symptoms	FHVP (mmHg)	WHVP (mmHg)	HVPG (mmHg)
1	44	M	HBV+HCV	9	F4	14	Ascites, jaundice, fluctuant encephalopathy	9	29	20
2	67	M	HCV	6	F4	8	No liver decompensation	6	18	12
3	54	F	HBV	6	F1	8	No liver decompensation, severe malnutrition	5	9	4
4	42	M	HBV+ Alcohol	11	F4	19	Ascites, jaundice, fluctuant encephalopathy	11	30	19
5	72	M	Alcohol	8	F4	11	Ascites and peripheral edema	10	28	18

Abbreviations: *HBV*, *HCV *(Hepatitis virus B, C); *MELD *(Model of end stage liver disease); *FHVP *(Free hepatic venous pressure); *WHVP *(Wedge hepatic venous pressure); *HVPG *(Hepatic venous pressure gradient)

The insertion of the ICE catheter was possible into the right and middle, but not into the left hepatic vein. The parenchyma of the right hepatic lobe was entirely visualized using ICE from the right or middle liver vein. Depending on the anatomy of each single case, parts of the left lobe could also be visualized from the middle hepatic artery. The right lobe of the liver was extensively visualized in all patients and the study of the parenchymal structure and of focal lesions was performed also with the use of color Doppler imaging. The relationship of focal lesions to vascular structures (portal vein, hepatic vein and artery) and to major bile ducts was mapped. Finally, the gallbladder and the presence of ascites were also investigated.

**Figure 2:**
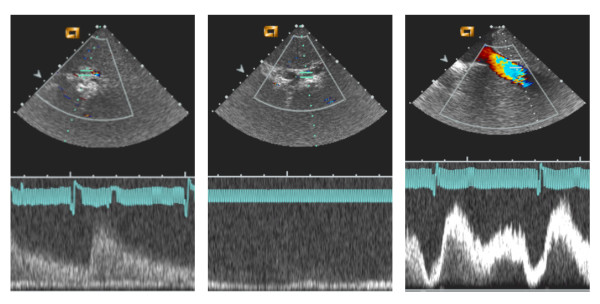
**Doppler imaging of the liver vasculature by intrahepatic use of an intracardiac echography catheter (ICE)**. The panel on the left shows the typical arterial wave (in this case corresponding to the right hepatic artery). The central panel corresponds to the right branch of the portal vein characterized by a continuous blood flow. The right panel illustrates the typical modulated flow of hepatic veins in the proximity of the confluence into the vena cava.

Liver vasculature was studied by ICE with the catheter tip inserted in the middle or the right hepatic vein. After adequate positioning, the hepatic artery or one of its branches was visualized and the flow was assessed using Doppler ultrasound in M-mode (7.5 MHz, peak flow 80 cm/s). Similarly, the portal vein and its branches (7.5 MHz, flow 15 cm/s), as well as the adjacent hepatic veins (7.5 MHz, variable flow) were studied using the same technique (Figure 2).

**Figure 3:**
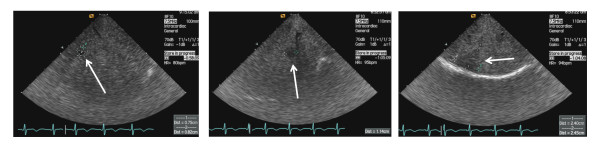
**Endovascular ultrasound view of focal lesions in a cirrhotic liver**. With high resolution the visualization of small sized nodules (diameter 0.82 cm, left panel, and 1.14 cm, central panel) was possible. The right panel shows a larger hypoechogenic lesion located in the sub-capsular region of a multinodular liver. Arrows indicate in each case the described lesions.

**Figure 4:**
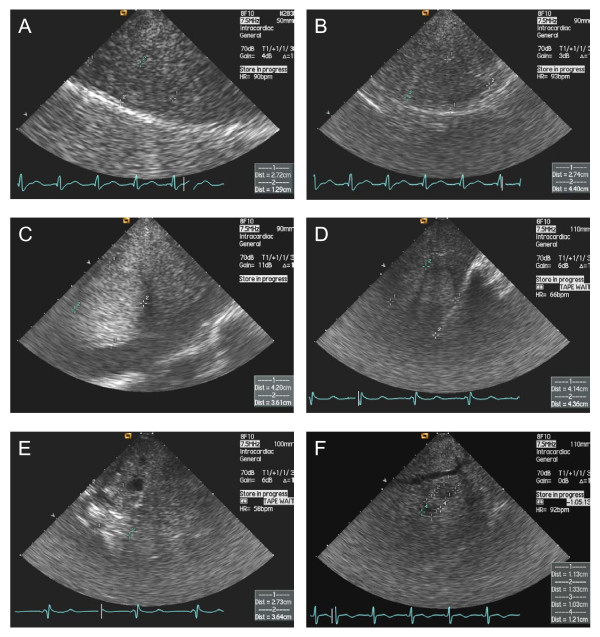
**Examples of focal lesions identified by ICE in patients with liver cirrhosis**. Without contrast medium for ultrasound, these tumors, which were highly suspect for hepatocellular carcinoma, could not be differentiated from regenerative nodules. The ultrasound device was introduced into the right or middle hepatic vein and the parenchyma surrounding the vessel was inspected over 360 degrees by turning the ultrasound catheter clock- and anti-clockwise. The ultrasound pattern of these lesions was homogeneous (**A**, **B **and **C**) or heterogeneous (**D**, **E **and **F**). No vascular invasion was identified in this study.

A selection of lesions that were identified in this study is presented in Figure 3 and data on single procedures and major findings are reported in Table 2. Additional images representing multiple macroscopic focal lesions are shown in Figure 4. All images were obtained with a frequency of the device of 7.5 MHz.

**Table 2:** Intravascular hepatic ultrasound characteristics

Patient	Duration (min)	Main findings	Complications
1	40	Heterogeneous hepatic parenchyma with nodular aspect	none
2	55	Single liver nodule, ascites	none
3	45	Multinodular aspect	none
4	45	Heterogeneous aspect of parenchyma, ascites	none
5	35	Hypovascular nodule in segment VII, ascites	none

The procedure of ICE was well tolerated and painless in all patients and the hemodynamic parameters remained stable during the entire intervention. There were no medical or technical complications during or after hepatic ICE. The catheterization of the right and middle hepatic vein was possible in all patients. Mostly due to unfavorable angle conditions and small diameter, ICE in the left hepatic vein was technically not possible.

**Figure 5:**
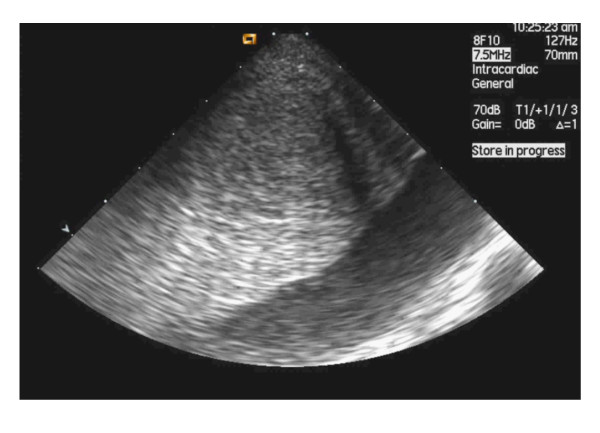
**Ascites in moderate quantity was visualized from the hepatic vein by intracardiac ultrasound technique**. The presence of an irregular hepatic border suggests nodular reorganization of the hepatic parenchyma as seen in cirrhosis.

Ascites was diagnosed in 3 of the 5 patients. Figure 5 shows an example of ascitic fluid collection in a patient with micro-nodular cirrhosis as suggested by the irregular liver surface.

The mean duration of this procedure was 44 minutes from the insertion to the extraction of the catheter.

## Discussion

This pilot study demonstrates the feasibility and safety of ICE for the evaluation of parenchymal alterations in patients with liver cirrhosis and suspected or proven hepatocellular carcinoma.

This technique is a novelty in the field of hepatology, but not in angiology and cardiology, where this tool is frequently used to guide non-surgical interventional procedures and to assess vascular disease [12,13]. High-resolution portal vein imaging has been the objective of a number of studies for evaluating possible invasion of the venous wall by pancreatic or ampullary cancers [14,15]. In other studies the liver has been investigated using intravascular ultrasound from the vena cava to guide the placement of direct intrahepatic porto-caval shunts in patients with portal hypertension [16,17]. However, studies aimed at examining liver parenchymal structure and lesions, as well as portal vein branches and hepatic veins are lacking. In this respect the present study is the first report on the use of this technique.

Beside the assessment of the feasibility and the safety, we also examined the imaging quality performance of ICE. By inserting the ICE catheter into the right and middle hepatic veins it was possible to visualize not only liver parenchyma, focal lesions and vascular features, but also extra hepatic structures such as the gallbladder and the peritoneal cavity to assess the presence of ascites. Due to the specific left hepatic vein anatomy and the size of the ICE catheter, we were not able to place the catheter into the left hepatic vein in order to obtain accurate images from this vessel. The failure to access the left hepatic vein represents an important limitation in cirrhotic patients, because a complete evaluation of the liver is essential for treatment planning. The use of ICE did however not present any other technical drawbacks.

At the end of the procedure patients were asked about the comfort and tolerability of ICE. The procedures were well tolerated, as demonstrated by the absence of pain and the hemodynamic stability throughout the procedure. It must be stressed however that we performed ICE as a complementary investigation following the measurement of HVPG. Hence, the invasiveness of ICE itself was possibly under estimated by the patients. The duration of ICE also represented a negative aspect, because it prolonged the patient's stay in the examination room. Finally, an additional radiation dose was used to guide and to verify the ultrasound catheter position. This supplementary irradiation might be avoidable in further studies by using conventional external ultrasound of the liver to follow the ICE device progression in the hepatic veins.

The performance of ICE in detecting nodules and visualizing blood vessels was tested qualitatively by comparing its findings with those obtained by using other imaging techniques such as CT or MRI in the same patients. Although the number of patients was small and the study was not designed to assess this endpoint, ICE findings were similar to those observed on images obtained by CT or MRI in the right hepatic lobe.

Future studies of ICE should assess the qualitative performance of this technique as a complement to external ultrasound, CT scan and magnetic resonance imaging for the evaluation of satellite small lesions and vascular invasion and/or thrombosis. Nevertheless, these results need first to be replicated in a larger number of patients before being translated into clinical strategies.

An additional point to be considered is the cost of this device, which is approximately 2500 Euros at this time. ICE catheters are for single use and their use must be accurately evaluated and be limited to clinical studies only. Besides diagnostic procedures, this technique might also be evaluated for interventional purposes, for example to assess the placement of transjugular intrahepatic porto-systemic shunts.

## Conclusions

This study demonstrated that a diagnostic approach using an ICE device is feasible in the right and middle hepatic veins, safe and well tolerated. However, it remains for the moment only an experimental investigative tool. This technique has the potential of an increased resolution power to image infra-centimetric vascular invasion. Whether ICE, as compared to other imaging techniques, could provide clinically relevant information in patients with parenchymal lesions and associated vascular alterations needs additional investigation.

## Abbreviations

ICE: intracardiac echography; HCC: hepatocellular carcinoma; HVPG: hepatic venous pressure gradient.

## Competing interests

The authors declare that they have no competing interests.

## Authors' contributions

ADG, EG, LS and AH designed this study. LS, PFK and ADG performed the procedures. ADG and LS drafted the manuscript. ADG, PFK, AH, EG and LS contributed to the interpretation of the results. ADG, PFK, AH, EG and LS critically reviewed the manuscript. ADG and EG took care of financial support. All authors read and approved the final manuscript.

### Availability of supporting data

The data set supporting the results of this article is included within the article.
